# Platysma motor branch transfer in brachial plexus repair: report of the first case

**DOI:** 10.1186/1749-7221-2-12

**Published:** 2007-05-02

**Authors:** Jayme Augusto Bertelli

**Affiliations:** 1 Department of Orthopedic Surgery, Governador Celso Ramos Hospital. Praça Getulio Vargas, 322, Florianópolis, SC, 88020030, Brazil

## Abstract

**Background:**

Nerve transfers are commonly employed in the treatment of brachial plexus injuries. We report the use of a new donor for transfer, the platysma motor branch.

**Methods:**

A patient with complete avulsion of the brachial plexus and phrenic nerve paralysis had the suprascapular nerve neurotized by the accessory nerve, half of the hypoglossal nerve transferred to the musculocutaneous nerve, and the platysma motor branch connected to the medial pectoral nerve.

**Results:**

The diameter of both the platysma motor branch and the medial pectoral nerve was around 2 mm. Eight years after surgery, the patient recovered 45° of abduction. Elbow flexion and shoulder adduction were rated as M4, according to the BMC. There was no deficit after the use of the above-mentioned nerves for transfer. Volitional control was acquired for independent function of elbow flexion and shoulder adduction.

**Conclusion:**

The use of the platysma motor branch seems promising. This nerve is expendable; its section led to no deficits, and the relearning of motor control was not complicated. Further anatomical and clinical studies would help to clarify and confirm the usefulness of the platysma motor branch as a donor for nerve transfer.

## Background

Nerve transfer, also called neurotization or nerve-crossing, consists of sectioning a normal nerve or branch and connecting its proximal stump to the distal stump of an injured nerve. This involves the sacrifice of a healthy nerve, the function of which should be compensated for by the remaining innervated muscles. This functional compensation can be promoted by simple agonist muscle hypertrophy or, when a partial denervation exists, through peripheral innervation from terminal axonal sprouting from intact adjacent motor units [1]. Nerve transfers are employed when a proximal nerve stump is not available for repair.

In brachial plexus reconstruction, available motor nerves for transfer originate either from the brachial plexus itself (i.e., intra plexual transfer) or extraplexually.

Among extraplexual branches already used are the accessory nerve, hypoglossal nerve, occipital nerve, cervical plexus, intercostals nerves, phrenic nerve, contralateral pectoral branches and contralateral C4 or C7 branches [2-4].

This paper reports for the first time the use of the platysma motor branch to reinnervate the pectoralis major muscle.

### Anatomical background of the cervical branch of the facial nerve

**Figure 1:**
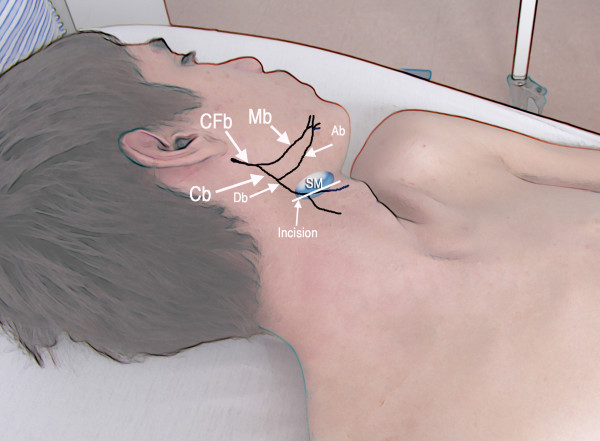
Schematic representation of the cervicomandibular branch of the facial nerve, its divisions and the surgical incision used to approach the platysma motor branch. (CFb) cervical branch of the facial nerve, which divides into the (Mb) mandibular branch and the (Cb) cervical branch. The Cb further divides into an (Ab) ascending branch, which is related to lower lip depression, and a (Db) descending branch, which innervates the lower portion of the platysma muscle. The Db is the branch used for transferring. (SM) submandibular gland.

Within the substance of the parotid gland, the facial nerve branches into the temporofacial and cervicofacial trunks. The cervicofacial division branches into the mandibular branch and the cervical branch. The cervical branch descends behind the ramus of the mandible, issues from the lower part of the parotid gland and runs anteroinferiorly under the platysma to the front of the neck to supply the platysma and communicate with the transverse cutaneous cervical nerve. In the suprahyiod region, the cervical branch follows a curve with superior concavity to travel forward along a course parallel and 3–4 cm distally to the lower border of the mandible [5,6]. The cervical branch divides into a branch to the anterosuperior portion of the platysma, which depresses the lower lip [7], and a branch to the lower portion of the muscle, which is the one used in the current case (Fig 1).

## Case presentation

A 21-year-old man sustained a right complete brachial plexus avulsion injury. Avulsion of all roots was confirmed by TCmyelo scan. Four months after trauma, under general anesthesia with the patient in the supine position, the brachial plexus was explored through a supraclavicular incision. All the roots were found to be avulsed and not graftable, and the phrenic nerve was paralyzed. The accessory nerve was transferred to the suprascapular nerve.

**Figure 2:**
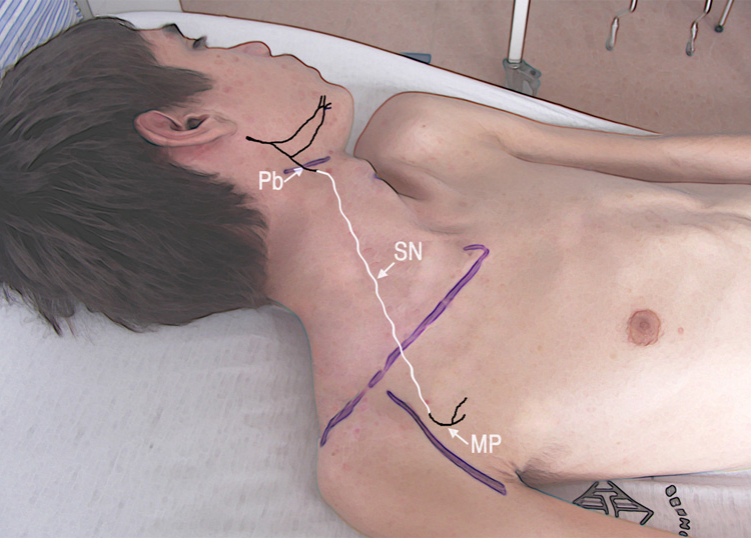
Schematic representation of the surgical procedure to connect the (Pb) platysma motor branch to the (MP) medial pectoral nerve. A (SN) sural nerve graft was used to connect donor and recipient nerves.

**Figure 3:**
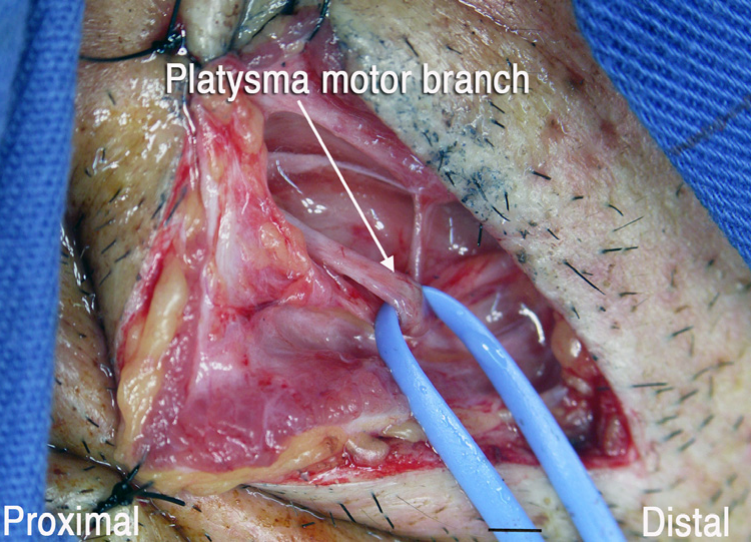
Intraoperative view of the platysma motor branch. Scale bar = 2 mm

**Figure 4:**
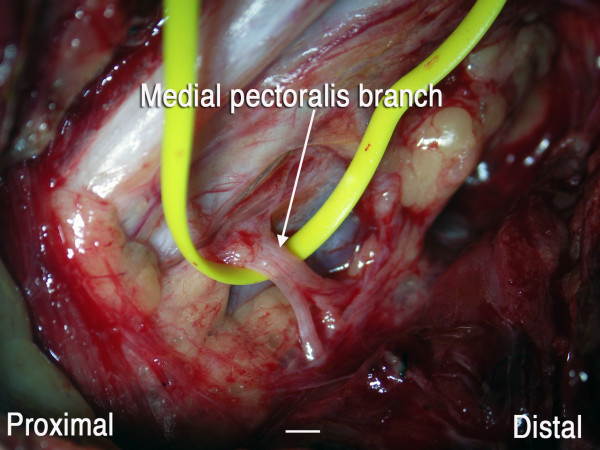
Intraoperative view of the medial pectoral nerve. Scale bar = 2 mm

A 5-cm incision was made 4 cm below the mandible, over the submandibular gland (Fig 1). The platysma muscle was divided and, immediately under it, the cervical branch of the facial nerve was identified. With the help of electric stimulation, the motor branch to the facial muscles (i.e., the ascending branch) was identified and preserved. Via this same incision, the submandibular gland was retracted cephalad, the hypoglossal nerve was dissected and sural nerve grafts were harvested. By a deltopectoral approach, the musculocutaneous and medial pectoral nerve were individualized. The hypoglossal nerve was hemi-sectioned and connected to the musculocutaneous nerve by means of a 22 cm sural nerve graft. The platysma motor branch was divided distally from the motor branch to the lip depressor muscles and connected to the medial pectoral nerve with a 20-cm sural nerve graft (Fig 2). The diameter of the platysma motor branch and the medial pectoral nerve was approximately 2 mm (Fig 3 and 4).

The patient was followed up regularly and, 8 years after surgery, had his final evaluation.

Two years after surgery, the patient had already recovery biceps and pectoralis major function. However, at this time, biceps contraction was clearly related to tongue motion. Five years after surgery, biceps activity was independent of tongue motion. Nevertheless, forced used of the tongue provoked biceps contractions. The patient referred that he first perceived pectoralis major activation during a forced deglutition. Contraction of the lower platysma muscle, but not lip depression, elicited pectoralis major activation. Five years after surgery, pectoralis major control was largely independent of platysma contraction. However, forced platysma contraction elicited pectoralis major co-contractions.

**Figure 5:**
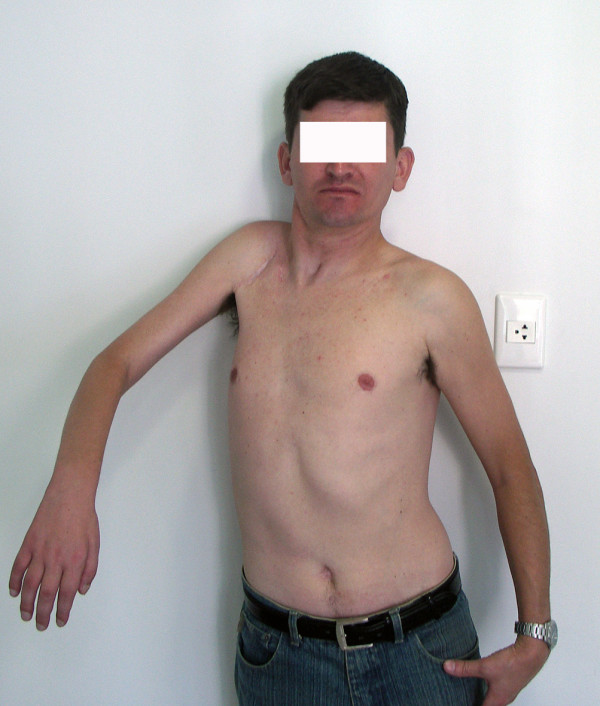
Results 8 years after surgery. The accessory nerve was connected to the suprascapular nerve, half of the hypoglossal nerve was grafted to the musculocutaneous nerve, and the platysma motor branch was transferred to the medial pectoral nerve. The patient recovered 45° of abduction and full elbow flexion, scoring M4. Shoulder adduction was restored with a M4 power. In 7, note shoulder adduction without concomitant elbow flexion. The independent control of these 2 functions is advantageous for the patient.

**Figure 6:**
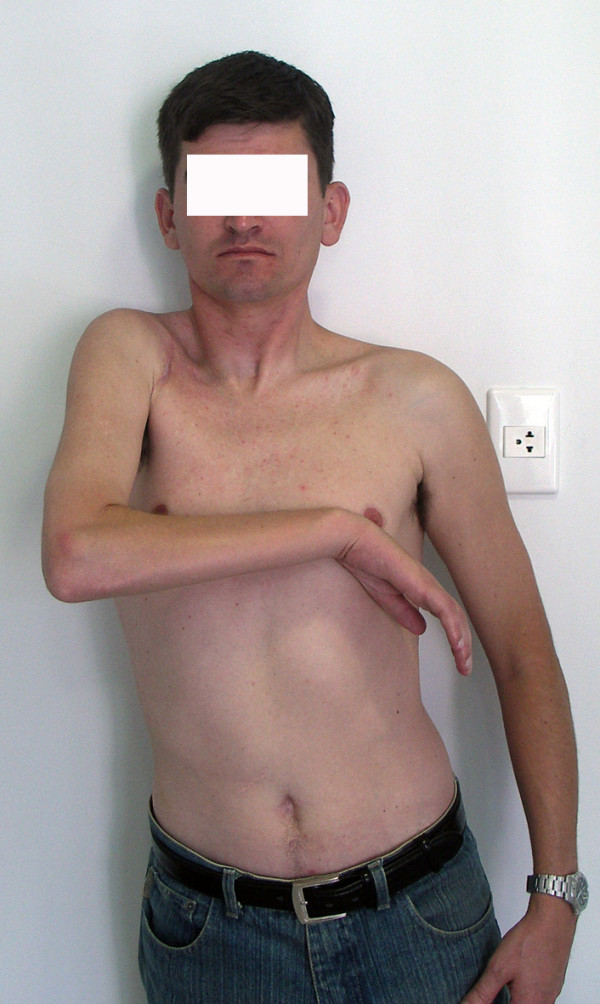
Results 8 years after surgery. The accessory nerve was connected to the suprascapular nerve, half of the hypoglossal nerve was grafted to the musculocutaneous nerve, and the platysma motor branch was transferred to the medial pectoral nerve. The patient recovered 45° of abduction and full elbow flexion, scoring M4. Shoulder adduction was restored with a M4 power. In 7, note shoulder adduction without concomitant elbow flexion. The independent control of these 2 functions is advantageous for the patient.

**Figure 7:**
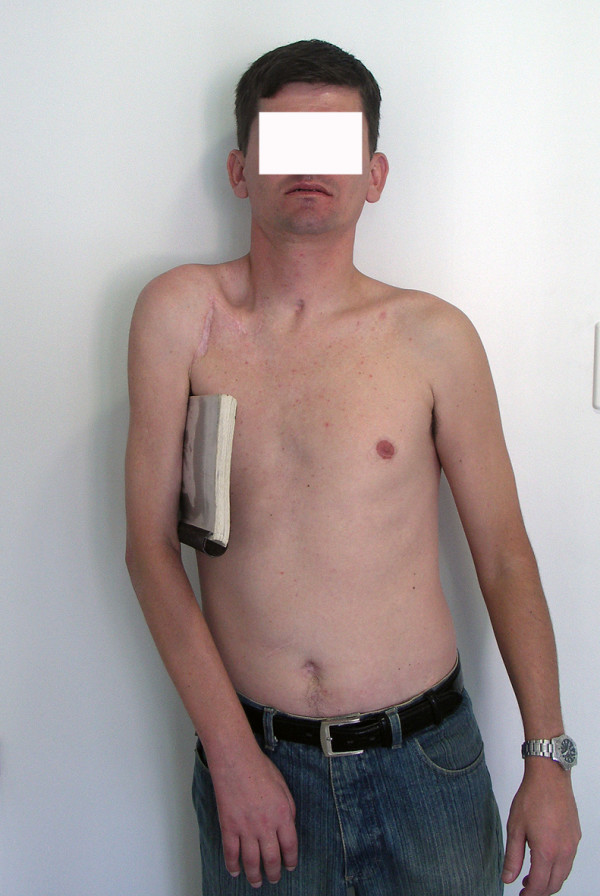
Results 8 years after surgery. The accessory nerve was connected to the suprascapular nerve, half of the hypoglossal nerve was grafted to the musculocutaneous nerve, and the platysma motor branch was transferred to the medial pectoral nerve. The patient recovered 45° of abduction and full elbow flexion, scoring M4. Shoulder adduction was restored with a M4 power. In 7, note shoulder adduction without concomitant elbow flexion. The independent control of these 2 functions is advantageous for the patient.

At the final evaluation, the patient had recovered 45° of abduction and antepulsion and complete elbow flexion. Elbow flexion and shoulder adduction strength were scored M4, according to the BMC system of evaluation. Only the sternal head of the pectoralis major muscle, which was reinnervated by the platysma motor branch, was functional. The patient was able to use his limb for assistance in daily activities and was capable of grasping things between the thorax and forearm and between the arm and the thorax. The patient could adduct the shoulder independently of the elbow flexion (Fig 5, 6, 7).

The nerve transfers were fully integrated, and there was no difficulty in reeducation.

Neither immediately after surgery nor in the long run were any deficits in the lip depressor function observed. There was no tongue atrophy and the platysma muscle remained functional.

## Discussion

In total brachial plexus palsy, the goal is to reconstruct at least 40° of abduction, shoulder adduction, and elbow flexion. There is no priority; all three of these functions should be reconstructed. In the sequence, the triceps long head is reinnervated as well as the wrist extensors, if sufficient donor nerves are available [8].

The present case demonstrated that, after total avulsion injury of the brachial plexus, a useful upper limb could be restored by neurotization of the suprascapular nerve, musculocutaneous nerve and medial pectoral nerve. It is important to isolate the function of the biceps and pectoralis major muscle to allow an object to be held within the arm and thorax without concomitant elbow flexion.

Preferably, the nerve transfer is connected to target nerves, rather than to nerve trunks, to avoid dispersion of the regenerating fibers with consequent failure. However, connecting nerve transfers to target nerves requires the use of long grafts. It has been suggested that, the longer the graft, the worse the results [9]. Millesi contends that the amount of nerve loss, rather than the length of the graft, contributes to impair the return of function [10]. It has been demonstrated that one possible reason for diminished recovery in long grafts is the increased rate of axonal misdirection[11], which might be counterbalanced by connecting a long graft to a single target muscle, similarly to what was done herein. In fact, clinicall studies revealed no difference in recovery in short grafts attached to the musculocutaneous nerve versus long grafts attached to the biceps motor branch [8].

The suprascapular nerve transfer to the accessory nerve is a standard procedure and the results herein obtained are in agreement with those from the literature [12]. Ferraresi et al [13] used a hemihypoglossal nerve transfer for musculocutaneous nerve reconstruction but – unlike the current results – gained no return of function. Mallessy et al [14] transferred the entire hypoglossal nerve to the musculocutaneous nerve and demonstrated biceps reinnervation. These authors did not observe volitional control of the nerve transfer, although they evaluated their patients for an average period of only 3 years, which may be a short interval for cortical integration of the hypoglossal to the musculocutaneous nerve transfer. The current study controlled the patient for 8 years and, initially, elbow flexion was dependent on tongue motion, but this dependence largely decreased over time. It is well known that some nerve transfers may take years for cortical integration and volitional control [3]. Malessy et al [14] employed the entire hypoglossal nerve and observed deficits in tongue motion. Like the results reported in Ferraresi et al [13], wherein only half of the hypoglossal nerve was used, our patient did not present tongue atrophy.

Pectoralis major muscle function was restored thanks to the transfer of the platysma motor branch. In this connection, the diameter of the platysma motor branch was around 2 mm and resembled that of the medial pectoral nerve. Volitional control of pectoral function was regained, probably because the cortical representation of the platysma muscle is not related to the facial muscles but, rather, is very close to hand function [15].

Even after sectioning of the platysma motor branch, platysma contraction was preserved, likely because of its supplementary innervation stemming from the cervical plexus [16]. Deficit following cervical branch lesion of the facial nerve can generate a pseudo-paralysis of the lip depressors that usually spontaneously recovers within 6 months, provided that the platysma muscle is not resected [17].

## Conclusion

The use of the platysma motor branch seems promising. This nerve is expendable, its section led to no deficits, and the relearning of motor control was not complicated. Further anatomical and clinical studies would help to clarify and confirm the usefulness of the platysma motor branch as a donor for nerve transfer.
